# A harmonized fast-fashion garment-variant dataset for textile circularity and sustainability assessment

**DOI:** 10.1016/j.dib.2026.113017

**Published:** 2026-06-23

**Authors:** Kai Li, Grit Walther

**Affiliations:** a Chair of Operations Management, RWTH Aachen University, Kackertstr. 7, 52072 Aachen, Germany; b Institute of Environmental Sciences (CML), Leiden University, 2333 CC Leiden, the Netherlands

**Keywords:** Material composition, Automated sorting, Fibre-to-fibre recycling, Component structure

## Abstract

Textile circularity research requires product-level data on garment composition and component structure, but most available information is either aggregated at fibre level, derived from physical audits with limited market coverage, or not organized at the colour-variant level needed for sorting and recycling assessment. This data article presents a harmonized fast-fashion garment-variant dataset compiled from publicly accessible Hennes and Mauritz (H&M) and Uniqlo product pages serving the United Kingdom and Australia.

Product web addresses (URLs) were collected from 24 to 26 March 2026, and product details were extracted between 24 March and 8 April 2026. Records were filtered, expanded to colour-specific variants where needed, harmonized into a common JSON Lines (JSONL) schema, normalized for material names and garment categories, parsed into component-level material-composition entries, and subjected to consistency checks. After filtering and public-release processing, the dataset contains 47,522 colour-specific garment-variant records. Each record includes source provenance, retailer and region, URL fields and timestamps, gender or section metadata, original category, product name, variant colour, assigned and normalized material-composition text, harmonized parent and detailed garment categories, and structured component-composition entries. The public release excludes product images, raw webpage captures, screenshots, review text, product ratings, review counts, and retailer-specific acquisition scripts.

The released files include the final JSONL dataset, processing scripts, mapping tables, processing summaries, citation metadata, license files, and workflow figures. Validation and quality-control information is provided through record-flow summaries, exported mapping tables, assignment-type diagnostics, component percentage-sum flags, and removal counts for unresolved or inconsistent records. The dataset can be reused for sustainability and circularity applications including sorting-compatibility assessment, pre-processing-rule development, design-for-recycling evaluation, fibre-to-fibre recycling analysis, material-flow modelling, life-cycle inventory construction, and comparative studies of online garment information.

Specifications Table

**Table:** 

Subject	Earth and Environmental Sciences
Specific subject area	Textile Circularity and Sustainability
Type of data	JSON Lines (JSONL) data; comma-separated values (CSV) mapping tables; plain-text (TXT) processing summaries; Python scripts; Portable Network Graphics/Scalable Vector Graphics (PNG/SVG) workflow figure; repository guide; citation metadata. Filtered; processed; harmonized; normalized; component-normalized; analysis-ready.
Data collection	Product web addresses (URLs) were collected from public Hennes and Mauritz (H&M) and Uniqlo listing pages for the United Kingdom and Australia from 24 to 26 March 2026. Product details were scraped between 24 March and 8 April 2026. Records were filtered, expanded to colour variants, harmonized, normalized by material and category, parsed into component-composition records, and prepared for public release.
Data source location	Product information was obtained from Hennes and Mauritz (H&M) and Uniqlo websites for the United Kingdom and Australia. The archived dataset is stored on Zenodo; workflow scripts and documentation are maintained in the associated GitHub repository.
Data accessibility	Repository name: Zenodo. Data identification number: 10.5281/zenodo.20006389. Direct URL to data: https://doi.org/10.5281/zenodo.20006389. Instructions for accessing these data: the final JSONL dataset and associated documentation, scripts, mapping tables, summaries, metadata, and license files can be downloaded from the Zenodo record [1]. The GitHub repository provides the maintained workflow documentation: https://github.com/kai-li-1994/garment-variant-dataset.
Related research article	None

## 1 Value of the Data

• The dataset records garments as colour-specific variants across two retailer websites and two regional portals. This structure preserves variant-level differences in colour and disclosed material composition that would be obscured if product pages alone were used as observations.
• The records link retailer-disclosed composition text to normalized material labels, harmonized garment categories, and parsed component structures. Researchers can therefore analyse fibres together with linings, fillings, trims, pockets, coatings, panels, decorative parts, and other components relevant to textile circularity.
• The data are suitable for developing and testing rule-based or model-based indicators for sorting compatibility, pre-processing needs, fibre-recovery constraints, recycling barriers, design-for-recycling attributes, garment complexity, and potential recycled-content claims.
• The release includes numbered Python scripts, comma-separated values (CSV) mapping resources, and plain-text (TXT) processing summaries that document the route from cleaned retailer records to the final component-normalized JSONL dataset. These files support reproducibility and adaptation to other retailer or market datasets.
• The dataset can inform industrial ecology applications including material-flow analysis, life-cycle inventory development, circular-economy scenario modelling, sustainability assessment, and policy-oriented monitoring where garment-level composition and component information are required but physical audit data are not available at sufficient scale.
• The dataset can also serve as a benchmark for future web-derived garment datasets, physical garment audits, post-consumer textile-waste characterization, sorting-validation studies, and studies of how online product disclosure can support supply-chain transparency and circularity assessment.

## 2 Background

Textile circularity research increasingly requires product-level information beyond aggregate fibre shares. Garments combine fibres, blends, linings, trims, coatings, pockets, fillings, decorations, and other components, all of which can influence sorting, dismantling, recycling-route suitability, and material recovery [2]. These features are particularly relevant because textile waste frequently contains blended fibres and multi-component products, which complicate automated identification, separation, and recycling [3].

Existing textile data sources only partly address this need. Physical garment audits and post-consumer textile-waste characterizations provide direct evidence on actual garments, but they are labour intensive and often limited in sample size, time, geography, or retailer coverage. Aggregate sustainability reports and fibre-flow datasets can support sector-level analysis, but they usually do not preserve garment-level colour, component, and composition information. Retailer product pages provide a complementary source because they commonly disclose product names, categories, colours, material-composition statements, and sometimes component-specific composition information [4].

The contribution of this dataset is therefore not to replace physical audits or official textile statistics, but to convert heterogeneous retailer-disclosed information into a harmonized, variant-level research resource. The article describes the dataset, file organization, construction workflow, field definitions, normalization resources, quality-control logic, and reuse context so that the dataset can be used independently for textile circularity, sorting, pre-processing, fibre-to-fibre recycling, material-flow analysis, life-cycle inventory development, and design-for-recycling studies.

## 3 Data Description

### 3.1 Overview of the dataset

The dataset consists of colour-specific garment variants obtained from online product pages of Hennes and Mauritz (H&M) and Uniqlo for the United Kingdom and Australia. Men’s, women’s, and children’s clothing are included. The final public data file is 6_JSONL_component_normalized_public.jsonl, archived in the Zenodo record. It provides harmonized garment-level information for studies of garment construction, textile-sorting constraints, pre-processing requirements, and fibre-to-fibre recycling disruptors. Product URLs were collected between 24 and 26 March 2026, and product-detail extraction took place between 24 March and 8 April 2026. To limit temporal drift, the URL set was fixed before detailed scraping. The records were then processed through variant expansion, schema harmonization, material-name normalization, garment-category normalization, and component-level parsing.

### 3.2 Repository and release structure

The GitHub repository is used as a maintained workflow record: it contains the scripts, mapping resources, summaries, workflow figure, citation metadata, and licenses associated with dataset construction. The Zenodo record provides the archived release package, including the final public JSONL file, 6_JSONL_component_normalized_public.jsonl, and the public-release summary, 6_JSONL_component_normalized_public_summary.txt. Table 1 lists the principal files distributed with the repository and archived release.

**Table 1:** File inventory of the GitHub repository and Zenodo release.

File	Description
README.md	Short repository guide with dataset access information, workflow-file orientation, citation information, and license notes.
6_JSONL_component_normalized_public.jsonl	Final public garment-variant dataset. This file contains 47,522 colour-specific garment variants and is included in the Zenodo release.
workflow_dataset_construction.png	Static workflow schematic showing how initial collected product-page records were transformed into the final dataset.
workflow_dataset_construction.svg	Editable vector version of the workflow schematic.
1_JSONL_drop_empty_summary.py	Script for filtering initial collected product-page records with missing material or colour information and reporting retained/dropped records by brand and region.
2_JSONL_uniqlo_variants_expansion.py	Script for expanding Uniqlo product-page records into colour-specific garment-variant rows and assigning material-composition text to each variant.
3_JSONL_key_harmonization.py	Script for harmonizing cleaned H&M records and expanded Uniqlo records into a shared JSON Lines (JSONL) schema.
4_JSONL_material_normalization.py	Script for normalizing material names and adding raw_material_text_norm while preserving original material fields.
5_JSONL_category_normalization.py	Script for assigning normalized garment categories and filtering out accessories, footwear, and unallocated records.
6_JSONL_component_normalization.py	Script for parsing material-composition text into structured component-level composition records and applying component consistency checks.
4_material_normalization_table.csv	Exported material-name mapping table derived from the canonical material groups and multilingual aliases used in the material-normalization script.
5_category_mapping_table.csv	Human-readable category mapping table linking rule order, parent categories, detail categories, include/exclude rule groups, and readable/raw patterns.
5_category_regex_table.csv	Technical regex inventory used for category assignment.
6_component_name_summary_table.csv	Summary table of normalized component names, component classes, and matched counts.
6_component_rule_mapping_table.csv	Full component-normalization rule mapping table containing regex patterns, readable rules, normalized component names, component classes, and matched counts.
1_JSONL_drop_empty_summary.txt	Processing summary for minimum-information filtering by brand and region.
2_uniqlo_JSONL_uk_cleaned_variants_summary.txt	Processing summary for Uniqlo UK colour-variant expansion. The same script was also run for Uniqlo Australia by changing the input file.
3_JSONL_harmonized_summary.txt	Processing summary for cross-retailer schema harmonization and harmonized line counts.
4_JSONL_material_normalization_summary.txt	Processing summary for material-name normalization, including row counts and number of changed normalized material-text records.
5_JSONL_category_normalized_summary.txt	Processing summary for category normalization, scope filtering, parent/detail category counts, and rule-source counts.
6_JSONL_component_normalized_summary.txt	Processing summary for component normalization, row filtering, component-class counts, component-name counts, and diagnostics.
LICENSE.txt	MIT license for source code and scripts.
LICENSE-DATA.md	Creative Commons Attribution 4.0 International (CC BY 4.0) statement for dataset files, derived tables, documentation, and metadata, unless otherwise stated.
CITATION.cff	Machine-readable citation metadata for GitHub and Zenodo citation support.
software_environment.txt	Software-environment note listing the programming language, package dependencies, and versions used for the final workflow execution.

The released package emphasizes the processed research dataset and the reproducible workflow rather than a redistribution of complete retailer webpages. Original scraped webpage records are not included because they may contain third-party website material that is unnecessary for scientific reuse, including image metadata, page-specific fields, review-related data, and other commercial webpage content. Transparency is instead provided through the processing scripts, mapping tables, and summaries that document how the public JSONL dataset was created.

Record flow across the construction workflow

Table 2 reports the change in record counts from the initial collected product-page records to the final public garment-variant dataset.

**Table 2:** Record flow across dataset construction steps.

Step	Input records	Removed or unresolved records	Output records	Main purpose
Initial collected records	47,834	264 removed	47,570	Remove records missing minimum material or colour information.
Uniqlo AU colour-variant expansion	1103 cleaned Uniqlo AU records	7 unresolved expanded rows	3081 variant rows	Convert Uniqlo AU product-page records into colour-specific variants.
Uniqlo UK colour-variant expansion	1490 cleaned Uniqlo UK records	16 unresolved expanded rows	3936 variant rows	Convert Uniqlo UK product-page records into colour-specific variants.
Cross-retailer harmonization	44,977 H&M records + 7017 Uniqlo variant records	0	51,994	Align H&M and Uniqlo records into a common schema.
Material-name normalization	51,994	0	51,994	Add canonical material labels while preserving original material text.
Category normalization and scope filtering	51,994	3750 removed	48,244	Assign harmonized garment categories and remove out-of-scope records.
Component normalization and consistency filtering	48,244	722 removed	47,522	Parse component-level composition and remove inconsistent records.
Final public dataset	47,522	—	47,522	Public JSONL dataset for textile circularity, sorting, preprocessing, and fibre-to-fibre recycling analyses.

### 3.3 Key fields in the final JSONL dataset

Table 3 defines the main record-level variables in 6_JSONL_component_normalized_public.jsonl.

**Table 3:** Main fields in the final JSONL dataset.

Field	Description
parent_product_id	Retailer product identifier for the parent product or product page.
Brand	Retailer brand, either hm or uniqlo.
Region	Retailer website region.
url	Product-page URL retained for provenance.
url_collected_at	Timestamp when the product URL was collected.
scraped_at	Timestamp when product details were collected.
gender_section	Retailer gender or section label.
raw_category	Original retailer category information. For H&M this may contain breadcrumb-style category information; for Uniqlo it is generally broader.
product_name	Retailer product name.
variant_colour	Colour label of the garment variant.
all_colour_labels	All colour labels listed for the parent product.
raw_material_text	Material-composition text assigned to the specific colour variant.
raw_material_text_full	Full raw material-composition text from the source record before variant-specific assignment.
composition_assignment_type	Method used to assign material-composition information to the colour-specific variant.
raw_description_text	Retailer-facing product description text retained where available.
raw_function_text	Retailer-facing function or attribute text retained where available.
raw_material_text_norm	Material-composition text after material-name normalization.
detail_category	Harmonized detailed garment category.
detail_rule_source	Field source used for category assignment, such as category tags, product name, or description text.
detail_rule_hit	Category rule that triggered the detail-category assignment.
parent_category	Harmonized parent garment category.
components_structured	Parsed and normalized component-level material-composition records.

## 4 Experimental Design, Materials and Methods

**Fig. 1:**
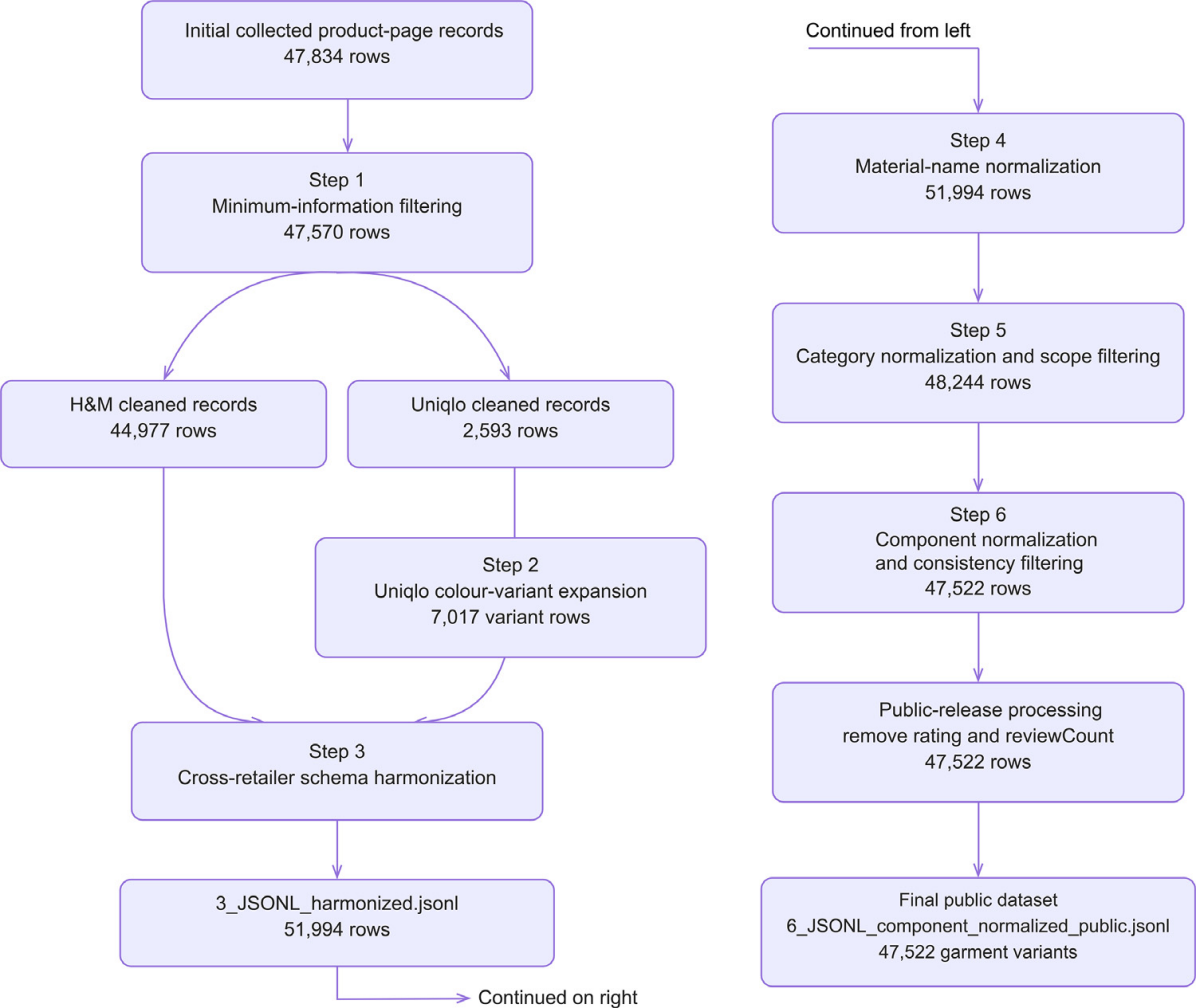
Workflow used to convert retailer product-page records into the public garment-variant dataset.

Fig. 1 gives an overview of the conversion from retailer product-page records to the public garment-variant dataset. The workflow covers minimum-information filtering, Uniqlo variant expansion, cross-retailer schema alignment, material-name normalization, garment-category normalization with scope filtering, component normalization, and public-release processing.

The methodological logic was to transform heterogeneous retailer disclosures into a stable analytical schema while preserving the original text fields needed for traceability. The workflow therefore separates source preservation from derived analytical fields: raw product names, categories, colours, and material-composition text are retained, while normalized material names, harmonized garment categories, and parsed component structures are added as reproducible rule-based outputs.

This structure was chosen instead of a purely aggregate fibre-share database because sorting, pre-processing, and fibre-to-fibre recycling decisions can depend on colour variants, linings, fillings, trims, pockets, coatings, panels, and other component-level features. It was also chosen instead of a manual physical-audit design because online product pages enable larger cross-market product coverage and can be updated or extended to other retailer portals. The trade-off is that the dataset reflects disclosed online information rather than measured physical garment composition; this limitation is addressed through provenance fields, retention of raw material text, deterministic mapping rules, and explicit consistency filtering.

### 4.1 Data source scope and product-page collection

The source data were obtained from publicly accessible online product pages on Hennes and Mauritz (H&M) and Uniqlo websites for the United Kingdom and Australia. H&M and Uniqlo were selected because their parent companies, H&M Group and Fast Retailing [5], are among the largest global apparel retailers after Inditex/Zara and because their websites provide structured product information suitable for scalable garment-level data construction. The United Kingdom and Australia portals were selected to introduce Northern and Southern Hemisphere seasonal contrast while keeping the retailer-region scope manageable. The dataset should therefore be understood as a structured product-level resource for two major fast-fashion retailers in two contrasting regional markets, not as a statistically representative sample of the entire fast-fashion sector. Brand names are used only to identify the source websites; the dataset is not affiliated with, endorsed by, or sponsored by H&M, H&M Group, Uniqlo, or Fast Retailing.

The clothing scope covers men’s, women’s, and children’s garments. Footwear, accessories, and unresolved non-garment categories were identified during category normalization and excluded from the category-normalized export. The final public dataset is therefore restricted to garments relevant to textile sorting, pre-processing, recycling, and textile circularity assessment.

Product web addresses (URLs) were collected from publicly accessible product-listing pages and associated listing endpoints for each retailer-region website and in-scope clothing category. The resulting URL set represents the products returned by the retailer listing pages at the time of collection. Retailer-specific acquisition scripts, endpoint-calling details, raw HyperText Markup Language (HTML), screenshots, and image-related metadata are not redistributed. URL collection occurred from 24 to 26 March 2026, and product-detail scraping was completed between 24 March and 8 April 2026. Because the URL pool was fixed before product-detail extraction, the later scraping step was applied to a predefined product set rather than to a continuously changing catalogue. The fields url_collected_at and scraped_at are retained so that users can distinguish URL capture from detail extraction.

The unit of analysis is a colour-specific garment variant. This choice reflects two data and reuse considerations: colour can matter for textile-sorting assessment, and material composition may differ between colour variants within the same nominal product. H&M records were treated as variant-level observations because the collected data represented different colours through distinct product-page URLs. Uniqlo pages, by contrast, could list several colours and sometimes multiple product identifiers or composition branches in one raw material field. The Uniqlo expansion script therefore created one row for each listed colour. When no branching was detected, a shared composition text was assigned to all colours. When branching was present, the script isolated product-identifier-specific text where available and used bracketed colour labels to assign composition text to individual colours. Unresolved assignments were dropped rather than inferred.

### 4.2 Dataset construction workflow

The construction workflow was implemented with numbered Python scripts and executed using Python 3.12.11 (conda-forge distribution) and pandas 3.0.2. The scripts also use Python standard-library modules including json, re, csv, pathlib, and collections. The workflow inputs and outputs are plain-text JSON Lines (JSONL), comma-separated values (CSV), and text (TXT) files. The software-environment note in the release reports these versions and can be used together with the numbered scripts to support reproducibility.

#### 4.2.1 Step 1: minimum-information filtering

The first workflow step removed records that lacked the minimum information required for subsequent processing. For H&M, the required fields were material_sum and colour_label. For Uniqlo, the required fields were fabric_details_raw and colour_labels. The initial collection contained 47,834 product-page records. Filtering removed 264 records and retained 47,570: 15,454 H&M Australia records, 29,523 H&M Great Britain records, 1103 Uniqlo Australia records, and 1490 Uniqlo United Kingdom records. The corresponding summary file is 1_JSONL_drop_empty_summary.txt.

#### 4.2.2 Step 2: uniqlo colour-variant expansion

The script 2_JSONL_uniqlo_variants_expansion.py converted Uniqlo page-level records into colour-specific variant rows. The same script was applied separately to each Uniqlo regional input by changing the input file.

For Uniqlo United Kingdom, 1490 cleaned records produced 3952 expanded rows before unresolved mappings were removed; after dropping 16 unresolved rows, 3936 variant rows remained. For Uniqlo Australia, 1103 cleaned records produced 3088 expanded rows before unresolved mappings; after dropping 7 unresolved rows, 3081 variant rows remained. Across both regions, Uniqlo contributed 7017 colour-specific variant rows to the harmonized dataset.

The field composition_assignment_type records the assignment logic for each variant. Table 4 formally documents all values generated by the script: native_variant_hm, shared_no_variants, mapped_by_colour_only, mapped_by_id_only, mapped_by_id_then_colour, mapped_by_other_colours_default, unresolved_colour_mapping, and unresolved_mapping. Unresolved cases were excluded from the expanded Uniqlo outputs rather than inferred.

**Table 4:** Composition-assignment types generated during Uniqlo colour-variant expansion.

Value	Meaning
native_variant_hm	H&M record was already treated as a colour-specific variant because colour variants were represented by distinct product-page URLs.
shared_no_variants	No composition branching was detected in a Uniqlo record; the same material-composition text was assigned to all listed colour variants.
mapped_by_colour_only	Composition was assigned using colour labels in the raw composition field.
mapped_by_id_only	Composition was assigned using product-identifier-specific information.
mapped_by_id_then_colour	Product-identifier-specific composition information was first isolated, then assigned by colour.
mapped_by_other_colours_default	A default “other colours” composition segment was assigned.
unresolved_colour_mapping	Colour-specific mapping could not be resolved; these rows were removed.
unresolved_mapping	General mapping could not be resolved; these rows were removed.

#### 4.2.3 Step 3: cross-retailer schema harmonization

The script 3_JSONL_key_harmonization.py merged cleaned H&M records and expanded Uniqlo records into a common JSON Lines (JSONL) schema. This step reconciled differences in the retailer source structures and retained only fields needed for provenance, garment description, material composition, category assignment, component parsing, and selected descriptive metadata.

The harmonized schema includes product and source identifiers, retailer region, URL, collection and scraping timestamps, gender or section metadata, original category, product name, colour information, assigned material-composition text, full source material text, composition-assignment method, description text, function or attribute text, and two consumer-engagement fields retained only before public-release processing. The harmonized output, 3_JSONL_harmonized.jsonl, contains 51,994 records: 44,977 from H&M and 7017 from Uniqlo.

#### 4.2.4 Step 4: material-name normalization

The script 4_JSONL_material_normalization.py added standardized material terminology by mapping abbreviations, brand-associated labels, multilingual labels, and synonymous material names to canonical terms. Examples include nylon/polyamide, elastane/spandex/lycra, polyester/PES/PET, cotton/supima, viscose/rayon, and wool/merino.

The source fields raw_material_text and raw_material_text_full remain unchanged. A separate field, raw_material_text_norm, stores the normalized text used in subsequent parsing. This design preserves the retailer-disclosed composition statement while making downstream material recognition more consistent.

The file 4_material_normalization_table.csv is generated from the canonical material groups and multilingual aliases implemented in the script. Material normalization processed 51,994 records and changed the normalized material-text field in 9392 records.

Material-name normalization was intentionally conservative. The script handles common synonyms, abbreviations, brand-associated material labels, selected multilingual aliases, and spelling variants explicitly included in the mapping table, while preserving raw_material_text and raw_material_text_full unchanged. It does not attempt probabilistic typo correction or language translation beyond the listed rules; unrecognized material wording remains visible in the retained source text and can be reviewed by future users. Table 5

**Table 5:** Examples of material-name normalization.

Normalized material name	Example raw labels mapped to this term
nylon	nylon; polyamide; pa; pa6; pa66
polyester	polyester; pes; pet; repreve
elastane	elastane; spandex; lycra; elastaan
cotton	cotton; supima; katoen
wool	wool; merino; merino wool
viscose	viscose; rayon
lyocell	lyocell; tencel; tencel lyocell
polyurethane	polyurethane; pu
leather	genuine leather; cowhide; goatskin; sheepskin; lambskin; nappa
metal	metal and selected unspecified metal labels
unspecified_material	other material; other materials; other fibres; unknown; synthetic

#### 4.2.5 Step 5: category normalization and scope filtering

The script 5_JSONL_category_normalization.py assigned harmonized garment categories using a two-level taxonomy. The taxonomy was informed by retailer-native category information and by sorter-oriented grouping logic from the Sorting for Circularity Europe handbook [6]. The resulting retained parent categories are tops, bottoms, overall, and underwear, with more detailed child categories below them. Footwear, accessories, and unallocated records were treated as outside the retained analytical clothing scope.

For category assignment, the script built searchable fields from retailer category tags, product names, and product descriptions. Field priority differed by retailer: H&M category breadcrumbs were usually more informative, whereas Uniqlo product names often carried the clearest garment-category signal. Rules were applied deterministically in a fixed order, with early scope exclusions for footwear and accessories and with more specific garment rules evaluated before broad fallback categories.

Category normalization processed 51,994 records. It removed 3750 records outside the retained clothing scope or without allocation: 2648 accessories, 1087 footwear records, and 15 unallocated records. The 15 unallocated records were not structural parsing errors. They were source records that passed minimum material and colour filtering but did not match any footwear, accessories, or retained garment-category regex rule with sufficient confidence. They were therefore excluded to avoid assigning unsupported categories. The category-normalized output contains 48,244 records. The most frequent detailed categories are t-shirt/polo, dresses, sweater/cardigan, trousers, shirt/blouse, and outerwear jacket. Table 6

**Table 6:** Parent and detail garment categories.

Parent category	Detail categories
tops	outerwear_coat, outerwear_jacket, outerwear_gilet, shirt_blouse, tshirt_polo, tank_camisole_vest, sweater_cardigan, sweatshirt_hoodie, top_generic
bottoms	jeans, trousers, leggings, joggers, shorts, skirts
underwear	underwear_bottoms, bras_lingerie, swimwear, socks_hosiery
overall	dresses, jumpsuits_overalls, sleepwear_homewear, set
footwear	footwear, removed from the exported category-normalized clothing dataset
accessories	accessories, removed from the exported category-normalized clothing dataset

#### 4.2.6 Step 6: component normalization and consistency filtering

The script 6_JSONL_component_normalization.py converted normalized material-composition text into structured component-level entries. This step was needed because retailer composition statements may describe a single main material composition or multiple garment parts, such as shell, lining, pocket lining, body, rib, padding, lace, coating, trim, or other component labels.

The component parser extracted component-material blocks, material shares, and recycled-content shares where these were disclosed. It then assigned standardized component names and broader component classes with rule-based mappings. The resulting components_structured field is a list of component entries containing the raw component path, normalized component name, component class, material entries, percentage total, consistency flag, and raw component text.

Component normalization processed 48,244 category-normalized records. Four records without usable material text and 718 records with component percentage totals above the consistency threshold were removed. The consistency threshold was pct_sum > 102 for any parsed component block, allowing minor rounding above 100% while excluding records whose parsed component materials were clearly inconsistent. These over-threshold cases can occur in web-derived fashion data when retailer composition text combines repeated segments, alternative colour or product-identifier compositions, nested component labels, recycled-content notes, or non-compositional warnings in the same field. The final component-normalized dataset contains 47,522 records and 68,427 component occurrences. Surface components are the largest class, followed by lining, pocket, trim, panel, filling, other, and decoration components. Table 7, Table 8, Table 9

**Table 7:** Fields inside each components_structured entry.

Field	Description
component_path_raw	Raw component label from the material-composition string.
component_name_norm	Standardized component name assigned by the component-normalization rules.
component_class	Broader component class used for analysis and aggregation.
component_norm_source	Source of the component-normalization decision.
materials	List of material entries for the component.
pct_sum	Sum of reported material percentages for the component.
pct_sum_flag	Percentage-sum consistency flag.
raw_text	Component-level raw material-composition block.

**Table 8:** Fields inside each component-level material entry.

Field	Description
material	Normalized material name.
pct	Material percentage within the component.
recycled_pct	Reported recycled-content percentage where available; otherwise null.

**Table 9:** Component classes in the final dataset.

Component class	Count	Description and examples
surface_component	48,573	Main visible or surface textile layer, including main, shell, body, face, base_fabric, outer_layer, and coating.
lining_component	9621	Inner or concealed textile layers, including lining, body_lining, sleeve_lining, hood_lining, inner_layer, cup_lining, interlining, and petticoat.
pocket_component	4786	Pocket-related components, including pocket, pocket_lining, pocket_fabric, chest_pocket_fabric, and patch_pocket.
trim_component	2673	Local trims or edge/detail structures, including collar, cuff, rib, lace, elastic_part, waist, belt, binding, tape, piping, and strap.
panel_component	1348	Named garment panels or local fabric sections, including front_panel, back_panel, side_panel, bottom_panel, top_panel, sleeve, hood, panel, woven_part, and knit_part.
filling_component	876	Filling, padding, or insulation components, including filling, padding, body_filling, upper_body_filling, and under_body_filling.
other_component	375	Reviewed residual component labels that did not fit the analytical classes above, including mesh, middle layer, other, other fabric, inner_support, details, and storage_bag.
decoration_component	175	Decorative textile or surface-detail elements, including embroidery, frill, fringe, tulle, faux_fur, application, pattern_area, and decorating_thread.

### 4.3 Reuse context and comparison with related datasets

The dataset complements rather than replaces physical textile-anatomy and post-consumer textile-waste datasets. For example, physical garment audits can provide measured mass, label, construction, and waste-market information, while this dataset provides a larger web-derived product snapshot with colour-specific variant structure, retailer provenance, disclosed composition text, normalized categories, and parsed component fields. The Nordic textile anatomy database is an example of a Data in Brief dataset based on garment composition in retail and post-consumer textile contexts [7]; the present dataset differs by emphasizing scalable online product-page extraction, cross-retailer schema harmonization, colour-specific variant expansion, and component parsing from retailer-disclosed composition text.

Potential reuse pathways include linking variant-level material compositions to sorting rules, estimating the prevalence of multi-component structures, identifying garments likely to require pre-processing before recycling, building life-cycle inventory inputs for representative garment categories, comparing composition disclosure across brands or regions, and testing whether online product information can support future digital product passport or textile circularity indicators. The retained URLs and timestamps support provenance checking, while the raw and normalized fields allow users to either reuse the provided harmonization or apply alternative mapping rules.

### 4.4 Public release and licensing

The repository and Zenodo record distribute the processed research dataset and workflow documentation, not a full copy of the retailer webpages. The public JSONL file was produced by removing non-essential consumer-engagement fields from the component-normalized data while keeping the same 47,522 records. The removed fields were rating and reviewCount.

The release does not include product images, model images, screenshots, raw HTML, full webpage captures, direct image URLs, review text, retailer-specific acquisition scripts, or other webpage material not required for dataset reuse. Source code is released under the MIT License, while the dataset, derived tables, summaries, documentation, and metadata are released under the Creative Commons Attribution 4.0 International (CC BY 4.0) license unless otherwise stated. The data license does not apply to third-party retailer webpages or website content that is not redistributed.

## Limitations

The dataset is based on information disclosed on retailer product pages and should not be interpreted as a physical garment teardown dataset. Some garment features may be absent when they were not disclosed by the retailer or could not be inferred from product-page information. The data represent a fixed online product snapshot defined by URL-collection and scraping timestamps; product pages, product availability, prices, and composition statements may have changed after collection. The retailer and regional scope is limited to H&M and Uniqlo websites serving the United Kingdom and Australia, so the dataset should not be interpreted as statistically representative of all fast fashion, all apparel retailers, or all national markets.

Normalized material names, garment categories, and component classes are deterministic rule-based analytical constructs designed to make heterogeneous retailer information comparable. The scripts handle selected synonyms, abbreviations, spelling variants, multilingual aliases, retailer nomenclature, and component labels that were explicitly encoded in the mapping rules. They do not provide general typo correction, automated translation, or probabilistic interpretation of un-mapped text segments. Consequently, unusual spelling, regional terminology not covered by the rules, or ambiguous composition strings may remain only in the retained raw text or may be removed during consistency filtering. Users extending the dataset to other retailers, languages, or regions should inspect unmatched and filtered records and update the mapping tables accordingly.

To reduce redistribution of third-party website content, the public release excludes product images, raw HTML, screenshots, full webpage captures, review text, ratings, review counts, and retailer-specific acquisition scripts. Brand names are retained only for source attribution. The dataset supports reproducible assessment of potential textile circularity, sorting, and pre-processing indicators, but it does not directly measure industrial sorting performance, pre-processing efficiency, realised fibre-to-fibre recycling outcomes, consumer behaviour, or actual post-consumer waste composition.

## Ethics Statement

The authors have read and followed the ethical requirements for publication in Data in Brief. This work did not involve human subjects, animal experiments, clinical data, personal data, or data collected from social media platforms. The dataset was derived from publicly accessible retailer product pages and processed into a research dataset. Product images, raw HTML, screenshots, review text, ratings, review counts, and retailer-specific acquisition scripts are not redistributed in the public release. Brand names are used solely to identify source websites. The dataset is independent of H&M, H&M Group, Uniqlo, and Fast Retailing and is not affiliated with, endorsed by, or sponsored by these companies.

## CRediT Author Statement

**Kai Li:** Conceptualization, Methodology, Software, Data curation, Formal analysis, Investigation, Validation, Visualization, Writing – original draft, Writing – review and editing, Project administration; **Grit Walther:** Conceptualization, Methodology, Supervision, Funding acquisition, Writing – review and editing.

## Data Availability

ZenodoA harmonized fast-fashion garment-variant dataset for textile circularity and sustainability assessment (Original data).
